# Microalbuminuria indicates long-term vascular risk in patients after acute stroke undergoing in-patient rehabilitation

**DOI:** 10.1186/1471-2377-12-102

**Published:** 2012-09-24

**Authors:** Dirk Sander, Christian Weimar, Peter Bramlage, Tobias Brandt, Ludger Rosin, Mario Siebler

**Affiliations:** 1 Department of Neurology, Benedictus Krankenhaus Tutzing & Feldafing, Abteilung für Neurologie, Dr.-Appelhans-Weg 6, Feldafing, 82360, Germany; 2 Department of Neurology, Technische Universität München, Munich, Germany; 3 Department of Neurology, University of Duisburg-Essen, Hufelandstrasse 55, Essen, 45122, Germany; 4 Institute für Pharmakologie und präventive Medizin, Menzelstr.21, Mahlow, 15831, Germany; 5 Kliniken Schmieder Heidelberg GmbH & Co, KG Speyererhof, Heidelberg, 69117, Germany; 6 Sanofi Aventis Germany GmbH, Potsdamer Str. 8, Berlin, 10785, Germany; 7 Neurorehabilitation Center, Auf der Rötsch 2, Essen-Kettwig, 45219, Germany

## Abstract

**Background:**

Patients in neurologic in-patient rehabilitation are at risk of cardio- and cerebrovascular events. Microalbuminuria (MAU) is frequent and an important risk predictor but has not been validated in in-patient rehabilitation. We therefore aimed to examine MAU as an indicator of risk and predictor of vascular events in a prospective study.

**Methods:**

The INSIGHT (**IN**vestigation of patients with ischemic **S**troke **I**n neurolo**G**ic re**H**abili**T**ation) registry is the first to provide large scale data on 1,167 patients with acute stroke (< 3 months) that survived the initial phase of high risk and were undergoing neurologic in-patient rehabilitation. MAU was determined by dipstick-testing and correlated to baseline clinical variables (stroke-origin, functional impairment, co-morbidity, ankle-brachial-index, intima-media-thickeness) as well as vascular events after one year of follow-up. Comparisons were made with the *χ*^2^ or Mann–Whitney-*U* Test. Relative risks (RR) with 95% confidence intervals (CI) were estimated using log-binominal models. To evaluate the association between MAU and new vascular events as well as mortality, we calculated hazard ratios (HR) using Cox proportional hazard regression.

**Results:**

A substantial proportion of patients was MAU positive at baseline (33.1%). Upon univariate analysis these patients were about 4 years older (69 vs. 65 years; p < 0.0001), had a slightly higher body mass index (27.8 vs. 27.1 kg/m^2^; p = 0.03) and increased waist circumference (79.5 vs. 50.4% for women [p < 0.0001] and 46.8 vs. 43.2% for men [p = 0.04]) and twice as often had diabetes mellitus (41.8 vs. 20.1%; p < 0.0001). Patients with MAU had a similar NIH stroke scale score (median 3 vs. 3; p = 0.379) but had lower values on the Barthel Index (median 75 vs. 90; p < 0.001). They had higher rates of atrial fibrillation (RR 1.38; 95% CI 1.09-1.75), coronary artery disease (RR 1.54; 95% CI 1.18-2.00), heart failure (RR 1.70; 95% CI 1.10-2.60) symptomatic peripheral artery disease (RR 2.30; 95% CI 1.40-3.80) and atherosclerotic stroke etiology (53.7 vs. 35.4%; p < 0.0001). MAU was associated with an increased intima-media-thickness, decreased ankle-brachial-index and polyvascular disease (RR 1.56; 95%CI 1.31-1.99). The event rate after a median follow-up of 13 months was 6.7% for fatal or nonfatal stroke, 4.7% for death, and 10.9% for combined vascular events (stroke, MI, vascular death). The presence of MAU was predictive for vascular events during the following year (HR for total mortality 2.2; 95% CI 1.3-3.7; HR for cardiovascular events 2.3; 95% 1.2 - 4.4).

**Conclusions:**

INSIGHT demonstrated a significant association between MAU and polyvascular disease and further supports previous findings that MAU predicts cardio-/cerebrovascular events in patients recovering from ischemic stroke. This biomarker may also be used in patients during neurologic in-patient rehabilitation, opening a window of opportunity for early intervention in this patient group at increased risk for recurrent events.

## Background

Microalbuminuria (MAU) is an established marker for both renal and cardiovascular risk [1]. It has been also shown to be indicative of asymptomatic cerebral ischemic lacunar infarcts observed on neuroimaging [2]. The relationship between MAU and incident stroke has been investigated in a number of cross-sectional and prospective studies with a documented positive correlation between the level of MAU and severity of the event.[3-8].

MAU is also a frequent finding in several acute clinical conditions including stroke [9-11]. Turay et al. (52 patients with recent stroke) found, that patients with MAU after acute stroke scored lower on the Scandinavian Stroke Scale than patients without MAU, had a correlation between the daily excretion of albumin and the severity of neurological deficit on admission (r −0.48, p < 0.05), had a lower score on the Barthel Index on Day 90 (median 65 vs. 100, p < 0.01), and had a higher 90 day mortality rate as compared to patients without MAU (45.8% vs. 7.1%) [11]. Slowik et al. (50 patients with recent stroke) also found, in a logistic regression analysis, that MAU was an independent predictor of 1-year mortality after ischemic stroke (OR 6.0; 95%CI 1.3-27.7) [10]. Available data on the predictive performance of MAU in patients with recent stroke therefore are derived from studies with low patient numbers [9-11], short follow-up [11], and focussing on those within 24h of acute stroke [10,11]. None of them has considered patients in neurologic in-patient rehabilitation within the first 3 months after stroke. These patients differ from those in acute medical care as they already survived the initial period with a high risk for morbidity and mortality.

To our knowledge, the INSIGHT (**IN**vestigation of patients with ischemic **S**troke **I**n neurolo**G**ic re**H**abili**T**ation) registry is the first to provide large scale data on 1,167 patients with acute stroke (< 3 months) that survived the initial phase of high risk undergoing neurologic in-patient rehabilitation [12]. In this dataset we aimed to examine (1) the prevalence of MAU, (2) the association with polyvascular disease, and (3) the predictive value of MAU for further vascular events.

## Methods

### Design

A total of 1,167 consecutive adult patients in neurologic in-patient rehabilitation with acute ischemic stroke (< 3 months) were included into this prospective registry between May and September 2008 [12]. Patients with hemorrhagic stroke (n = 212) and those not able to provide written informed consent (severe aphasia [n = 88]; severe cognitive deficits [n = 58]; retraction of initial informed consent [n = 41]) were excluded. Ethical approval was obtained by the Ethics Committee of Technical University Munich (Klinikum Rechts der Isar) and patients provided written informed consent prior to inclusion.

### Data collection

Baseline characteristics, co-morbid disease, physical examination, laboratory results, prior cardiovascular events, neurologic assessment (TOAST classification, NIH-Stroke Scale, Rankin score and Barthel Index) and concomitant drug use were collected on a standardized case report form at admission to neurologic in-patient rehabilitation (study onset). Neurological impairment and the resulting handicap were scored on the National Institutes of Health Stroke Scale (NIH-SS) [13], the modified Rankin scale [14,15], and the Barthel Index [16]. Microalbuminuria was determined with reagent strips (Microalbustix®, Bayer Vital, Leverkusen, Germany). The Microalbustix® reagent stripes measure creatinine and albumin semi-quantitatively. Based on the recommendations of the manufacturer albumin was adjusted to creatinine concentration and MAU presence was calculated using the provided diagram. There were no patients on maintenance hemodialysis, peritoneal dialysis, and renal transplantation in our patient cohort.

A central structured follow-up interview via telephone was performed after a median follow-up of 13 months (12 to 14 months) by a person blinded to the results of the other investigations. If the patient was alive and able to provide information, all data were retrieved from himself. If the patient had died or not able to provide information (e.g. due to aphasia), family members were asked. The interviewer underwent a specific training before performing interviews. In case of death within the follow-up period the cause of death (myocardial infarction, stroke or others) and the date was recorded. For patients alive at one year it was determined whether they had any cardiovascular event (myocardial infarction, recurrent stroke or transitory ischemic attack - yes, no and unknown) including frequency but without a specific date. Events were only obtained via telephone and not validated against medical records of the treating physician. In addition medication, symptoms of stroke, cardiovascular risk factors and modified Rankin score were evaluated. This kind of a structured interview has been tested for validity and reproducibility [17].

### Vascular risk factors

Risk factors determined included the following: smoking status, duration of smoking, arterial hypertension (treatment with antihypertensive medication or documented blood pressure >140 mm Hg systolic or >90 mm Hg diastolic before admission), hyperlipidemia (treatment with lipid-lowering medication or total cholesterol level ≥ 200 mg/dl, diabetes mellitus (treatment with antidiabetic drugs or diagnosis of diabetes during hospital stay), body mass index, prevalent ischemic heart disease [IHD] (documented by previous myocardial infarction or angina pectoris, bypass surgery, or > 50% angiographic stenosis of ≥ 1 major coronary artery), cholesterol, and triglycerides.

### ABI and IMT measurements

Ankle-brachial index (ABI), duplex ultrasonography including intima-media thickness (IMT), plaques and degree of carotid stenosis were performed on site by trained personnel. The plaque sum score was calculated according to Crouse [18]. For calculation of the ABI, both the lower and higher systolic blood pressure in each leg was divided by the average systolic pressure in both arms (except in case there was a discrepancy of ≥ 10mmHg between the two arms, only the higher blood pressure of both arms was used). An ABI ≤ 0.9 using the lower systolic blood pressure in one leg was considered indicative of subclinical peripheral artery disease (PAD) [19].

For the determination of IMT, the common carotid arteries (CCA) were examined in anterior, lateral, and posterior planes as described previously [20]. Experienced investigators performed the duplex ultrasonography using a standardized study protocol. The ultrasound data were stored on digital media, were transferred to the neurovascular laboratory of the Department of Neurology. The measurements of IMT were performed as previously described in detail with the use of a computer-supported image analysis system (Sigmascan- Pro 5.0, SPSS) [20].

### Statistical analyses

A subsample of hospitals were monitored on-site for validity and completeness of data obtained. Data entry into a validated database was performed in duplicate using SAS 8.02. Categorical variables are presented as percentages with 95% confidence intervals (CI), and continuous variable as mean plus standard deviation (SD). Comparisons were made with the *χ*2 or Mann–Whitney-*U* Test. Relative risk (RR) with 95% CI was estimated using a univariate log-binominal regression model. For multivariable adjustment of RR ratios, variables being significantly different at baseline (see Table 1 with a p-value < 0.05) were considered: age, body mass index, diabetes, waist circumference, systolic blood pressure, total and HDL-cholesterol, the use of ACEi/ARBs, calcium-antagonists insulin, oral antidiabetic drugs, differences in the functional status, stroke subtype, coronary artery disease and symptomatic peripheral artery disease. To evaluate the association between MAU and new vascular events as well as mortality, we calculated Hazard ratios (HR) using a Cox proportional hazard regression model. The primary endpoint was overall mortality after 1 year; secondary endpoints were stroke, MI, and vascular mortality. A p-value <0.05 was considered statistically significant.

**Table 1:** Baseline characteristics at admission to in-patient rehabilitation and pharmacotherapy of patients with or without MAU

	**Total (n = 1,167)**	**MAU (+) (n = 386)**	**MAU (−) (n = 781)**	**p-value**
Age, mean years ± SD	66 ± 11.9	69 ± 11.1	65 ± 12.5	< 0.0001
Male gender (%)	58.1	55.7	59.4	0.33
Body Mass Index (mean kg/m2 ± SD)	27.5 ± 4.7	27.8 ±5.2	27.1 ±1.5	0.03
Diabetes (%)	34.6	41.8	20.1	< 0.0001
Hypertension (%)	79.3	80.2	78.9	0.09
Hyperlipidemia (%)	80.3	81.3	79.7	0.08
Smoking				
Current (%)	18.4	17.7	18.7	0.93
Previous (%)	38.8	39.2	38.7	0.93
mean packs*years ± SD	32.1 ± 26.4	33.1 ± 27.8	31.9 ± 27.1	0.65
Waist circumference (%)				
men (% ≥ 102 cm)	44.4	46.8	43.2	0.04
women (% ≥ 88 cm)	40.9	79.5	50.4	<0.0001
Blood pressure				
systolic (mean mmHg ± SD)	134 ± 17	136 ± 18	132 ± 17	0.017
diastolic (mean mmHg ± SD)	77 ± 11	77 ± 12	77 ± 10	0.75
Laboratory parameters (%)				
Total cholesterol (mg/dl)	182 ± 46	178 ±47	184 ± 44	0.06
HDL-cholesterol (mg/dl)	48 ± 15	44 ±13	49 ± 17	0.0002
LDL-cholesterol (mg/dl)	107 ± 17	107 ± 21	107 ± 15	0.91
Triglycerides (mg/dl)	146 ± 71	145 ± 73	149 ± 69	0.63
Pharmacotherapy				
Lipid lowering agents (%)	64.6	66.1	63.9	0.49
ARB/ACEi (%)	45.9	56.2	40.9	0.0002
Betablocker (%)	46.2	45.2	46.7	0.23
Calciumantagonists (%)	29.9	24.8	27.9	0.09
Insulin	5.6	8.2	4.2	0.0003
Oral antidiabetic drugs	14.3	23.3	9.8	0.0002
Functional assessment				
NIH Stroke Scale (median, range)	3 (0–25)	3 (0–20)	3 (0–25)	0.379
Barthel Index (median, range)	85 (0–100)	75 (0–100)	90 (0–100)	<0.0001
Origin of Stroke (TOAST)				
Large artery atherosclerosis	41.4	53.7	35.4	<0.0001
Cardioembolic	21.8	22	21.6	0.456
Small vessel disease	18.9	13.7	21.4	0.01
Other aetiology	4.5	2.4	5.5	0.234
Undetermined aetiology	13.4	8.2	16.1	0.006

*Legend:* n, number; *SD* standard deviation, *ARB* angiotensin receptor blockers, *ACEi*, angiotensin converting enzyme inhibitor, *NIH* National Institutes of Health *p = 0.003 vs. all patients.

## Results

### Prevalence and patient characteristics

Between the onset of acute stroke and study inclusion at admission to neurologic in-patient rehabilitation there was a mean delay of 0.9 ± 0.5 months. A substantial proportion of patients were MAU positive at baseline (33.1%). Upon univariate analysis these patients were about 4 years older (69 vs. 65 years; p < 0.0001), had a slightly higher body mass index (27.8 vs. 27.1 kg/m^2^; p = 0.03) and waist circumference (79.5 vs. 50.4% for women [p < 0.0001] and 46.8 vs. 43.2% for men [p = 0.04]) and twice as often had diabetes mellitus (41.8 vs. 20.1%; p < 0.0001) (Table 1 and 2). Further differences were an elevation of systolic blood pressure (136 vs. 132 mmHg; p = 0.017) and lower HDL-cholesterol values (44 vs. 49 mg/dl; p = 0.0002). ACE inhibitors and ARBs were more frequently prescribed in MAU positive patients (56.2 vs. 40.9%; p = 0.0002). Neurological assessment of patients revealed that those with MAU had lower values on the Barthel Index (median 75 vs. 90; p < 0.001).

**Table 2:** Comparison of patients with or without follow-up

	**With follow-up (n = 846)**	**Without follow-up (n = 321)**
Age, mean years ± SD	64.2 ± 11.1	73.7 ± 10.9*
Male gender (%)	58.2	56.2
Body Mass Index (mean kg/m2 ± SD)	27.1 ±4.1	27.9 ±5.1
Diabetes (%)	68.1	65.9
Hypertension (%)	78.1	77.7
Hyperlipidemia (%)	79.5	79.8
Smoking		
Current (%)	19.3	20.1
Previous (%)	36.5	36.8
mean packs*years ± SD	31.8 ±26.5	32.9 ±28.5
Waist circumference (%)		
men (% ≥ 102 cm)	48.1	47.1
women (% ≥ 88 cm)	79.4	78.2
Blood pressure		
systolic (mean mmHg ± SD)	135 ±14.2	133 ±15.2
diastolic (mean mmHg ± SD)	78 ±11.4	77 ±11.3
Laboratory parameters (%)		
Total cholesterol (mg/dl)	181 ± 46.3	183 ± 47.1
HDL-cholesterol (mg/dl)	46 ± 15.2	48 ± 14.2
LDL-cholesterol (mg/dl)	108 ± 39.8	106 ± 37.8
Triglycerides (mg/dl)	142 ± 70.4	141 ± 70.8
Pharmacotherapy		
Lipid lowering agents (%)	63.1	63.9
ARB/ACEi (%)	45.6	43.6
Betablocker (%)	44.7	46.7
Calciumantagonists (%)	26.5	25.9
Insulin	5.4	5.7
Oral antidiabetic drugs	14.3	14.2
Functional assessment		
NIH Stroke Scale (median, range)	3.0 (0–25)	3.0 (0–25)
Barthel Index (median, range)	86 (0–100)	87 (0–100)
Origin of Stroke (TOAST)		
Large artery atherosclerosis	40.9	40.8
Cardioembolic	22.1	21.9
Small vessel disease	18.5	18.8
Other aetiology	4.6	4.5
Undetermined aetiology	13.9	14.0

*Legend:* n, number, *SD* standard deviation, *ARB* angiotensin receptor blockers, *ACEi* angiotensin converting enzyme inhibitor, *NIH* National Institutes of Health, *p = 0.003 vs. all patients.

### Co-morbid disease conditions

**Figure 1:**
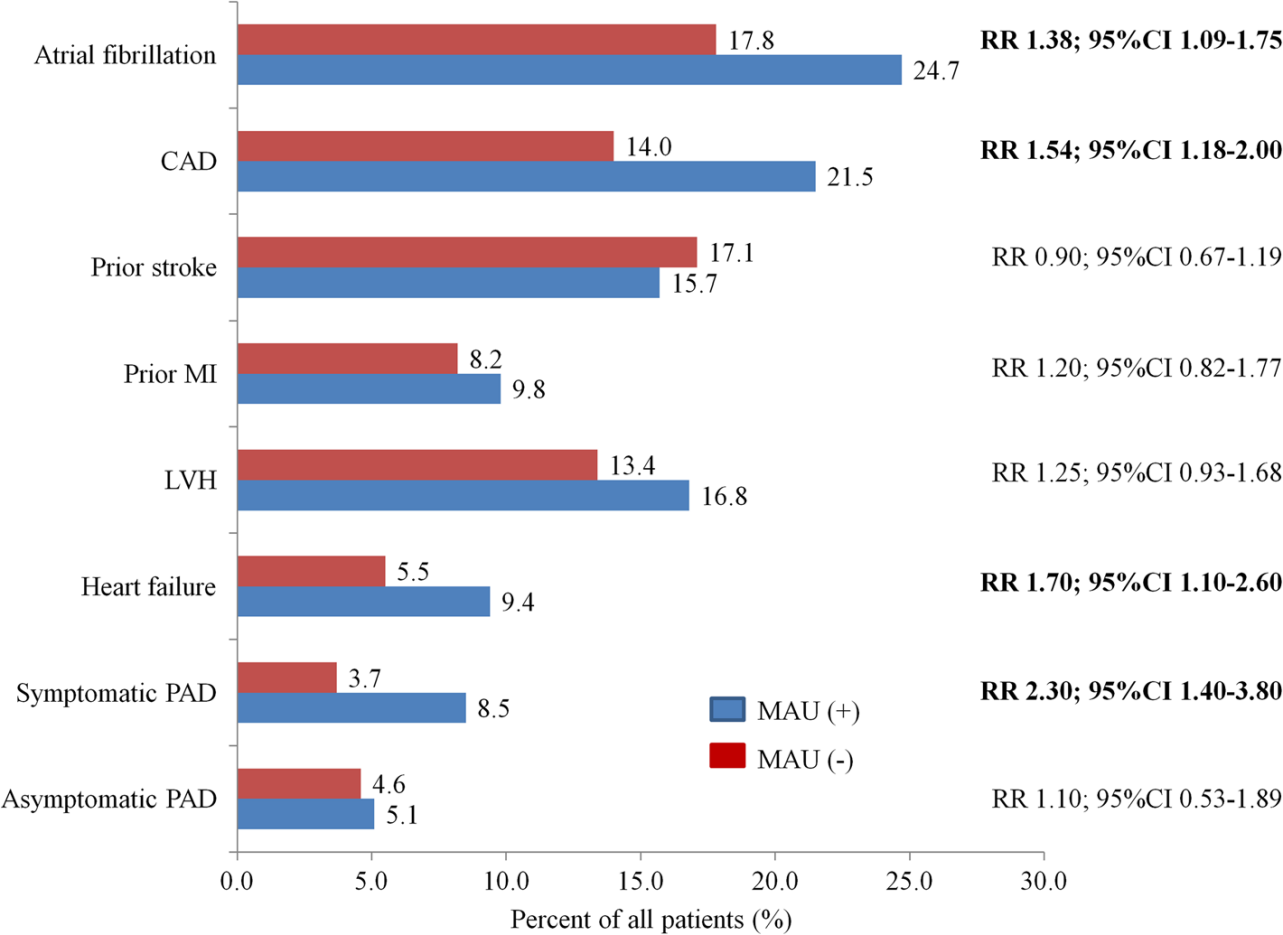
**Prevalence of co-morbid disease in patients with or without MAU (%)*Legend.*** MAU, microalbuminuria; PAD, peripheral arterial disease; RR, relative risk; CI, confidence interval.

Patients with MAU had higher rates of atrial fibrillation (RR 1.38; 95%CI 1.09-1.75), coronary artery disease (RR 1.54; 95%CI 1.18-2.00), heart failure (RR 1.70; 95% 1.10-2.60) and symptomatic peripheral artery disease (RR 2.30; 95%CI 1.40-3.80) compared to patients without MAU (Figure 1; unadjusted). MAU was closely correlated with an atherosclerotic origin of stroke (Table 1). In contrast, MAU positive patients showed a microangiopathic or an unknown cause of the stroke according to the TOAST classification less frequently.

### Correlations of MAU with ABI and IMT

Given the higher frequency of MAU in patients with an atherosclerotic origin of stroke, we aimed to assess whether there was a correlation between MAU and the extent of atherosclerosis in different vascular beds (Table 3). We observed that patients with MAU had an increased IMT and a decreased ABI (<0.9), even after adjustment for baseline variables and other parameters (defined in the legend to Table 3). The detection of MAU was independently associated with an increased prevalence of polyvascular disease (IMT > 1 mm and an ABI <0.9) with an RR of 1.56; (95%CI 1.31-1.99, p = 0.0001).

**Table 3:** **Association between MAU and atherosclerosis in different vascular beds (Adjusted for variables with significant differences (p < 0.05) in Table**1**)**

	**MAU (+) n = 386**	**MAU (−) n = 781 (ref.)**	RR (95%CI)*	**p-value**
*ABI (lower value of either ankle BP used)*				
≤ 0.9 (%)	31.5	23.8	1.32 (1.09-1.61)	0.004
<0.6 (%)	10.8	3.4	1.91 (1.55-2.37)	0.0002
0.6-0.9 (%)	20.7	20.4	1.01 (0.82-1.24)	0.884
> 0.9 - 1.5 (%)	66.1	72.6	0.82 (0.69-0.98)	0.027
>1.5 (%)	2.4	3.6	0.71 (0.40-0.72)	0.29
*CCA IMT (mm)*				
1. Quartile (< 0.75) (%)	14.5	24.5	0.63 (0.49-0.89)	0.0002
2. Quartile (0.75-0.85) (%)	22.5	19.0	1.13 (0.94-1.37)	0.148
3. Quartile (0.85-1.05) (%)	33.7	32.5	1.04 (0.87-1.23)	0.69
4. Quartile (>1.05) (%)	29.3	24.1	1.21 (1.00-1.42)	0.054
> 1mm (%)	31.1	24.7	1.24 (1.02-1.51)	0.029
*Polyvascular disease*				
IMT > 1 mm and ABI ≤ 0.9 (%)	13.3	6.1	1.56 (1.3-1.99)	0.0001

*Legend: ABI* ankle brachial index, *BP* blood pressure, *CCA* common carotid artery, *IMT* intima media thickness, *MAU* microalbuminuria, *RR* relative, * For multivariable adjustment of ORs, variables being significantly different at baseline (see Table 1 with a p-value < 0.05) were considered: age, body mass index, diabetes, waist circumference, systolic blood pressure, total and HDL-cholesterol, the use of ACEi/ARBs, calciumantagonists and differences in the functional status as well as stroke subtype (TOAST), coronary artery disease and symptomatic peripheral artery disease as outlined in the methods section.

### MAU testing in relation to vascular events up to 1 year

A follow-up was available in 846 patients (72.5%). Patients lost to follow-up were significantly older but not significantly different with regard to other baseline characteristics. The event rate after a median follow-up of 13 months was 6.7% for fatal or nonfatal stroke and 10.9% for combined vascular events (stroke, MI, vascular death). 40 out of 846 patients (4.7%) died during the one year follow up, the most frequent reasons being stroke (n = 9, 22.5%), malignancy (n = 9, 22.5%), myocardial infarction (n = 3, 7.5%), and heart failure (n = 3, 7.5%). Other causes were rare (n = 1). For 5 patients no information on the cause of death was available.

The clinical utility of MAU testing to independently predict vascular events is displayed in Table 4**.** It was shown that total (HR 2.2; 95%CI 1.3-3.7) and cardiovascular mortality (HR 2.5; 95%CI 1.4-4.7) as well as cardiovascular events such as stroke and myocardial infarction (HR 2.3; 95% 1.2-4.4) were increased in patients with MAU.

**Table 4:** Association between MAU testing and new vascular events using Cox proportional Hazard regression

**Events**	**MAU (+) n = 280**	**MAU (−) n = 566**	**HR (95%CI) †**	**p-value**
*Primary endpoint*				
Total mortality (%)	7.8	3.3	2.2 (1.3-3.7)	0.008
*Secondary endpoints*				
Cardiovascular mortality (%)	5.2	2.8	2.5 (1.4-4.7)	0.002
Cardiovascular events (%)*	8.2	4.1	2.2 (1.32-3-72)	0.003
Stroke (%)	6.1	3.2	2.1 (1.05-4.2)	0.004
Myocardial infarction (%)	2.1	0.9	2.3 (1.2-4.4)	0.008

*Legend:* * Stroke and/or MI; *MAU* microalbuminuria, *HR* Hazard ratio, *CI* confidence interval; † For multivariable adjustment of HRs, variables being significantly different at baseline (see Table 1 with a p-value < 0.05) were considered: age, body mass index, diabetes, waist circumference, systolic blood pressure, total and HDL-cholesterol, the use of ACEi/ARBs, calciumantagonists, insulin, oral antidiabetic drugs and differences in the functional status as well as stroke subtype (TOAST), coronary artery disease and symptomatic peripheral artery disease as outlined in the methods section.

## Discussion

The present analysis demonstrates that MAU is associated with a number of vascular risk factors and co-morbid disease conditions in patients undergoing neurologic in-patient rehabilitation after ischemic stroke. These patients already survived the initial high risk phase after stroke but remain at long-term risk for recurrent vascular events. We were able to show that MAU is a powerful indicator of atherosclerotic risk in this patient population and a predictor of vascular events and mortality within a median 13 months follow-up. These data reinforce the utility of MAU as a biomarker in clinical practice.

### Patient population and prevalence of MAU

Prior data on the prevalence of stroke have been reported by Beamer et al., who demonstrated that MAU was 3 times more prevalent in patients within 6–8 weeks after stroke (29%) compared with those without (10%) [9]. The prevalence of MAU reported by Slowik et al. in patients within 24h after acute stroke was 46.7% [10]. On admission, these patients had a more severe neurological deficit and more often had a decreased level of consciousness. This was well matched by Turaj et al. who reported a prevalence rate of 46.1% within 24h after acute stroke and found a correlation between the daily excretion of albumin and the severity of the neurological deficit (r = −0.48; p0.05) [11]. In our present analysis 33% of patients undergoing neurologic in-patient rehabilitation had MAU, which was, upon univariate analysis, associated with diabetes, age, a high waist circumference, increased systolic blood pressure and an increased impairment of the activities of daily living.

### Co-morbid disease conditions

Patients with MAU had higher rates of atrial fibrillation. This observation corroborates several previous studies that showed an association between MAU and an increased incidence of atrial fibrillation [21,22].On the other hand, MAU correlated less powerful to a cardioembolic origin of stroke. This finding may be explained by the fact that majority of patients with atrial fibrillation in our study took oral anticoagulants during the follow-up period.

Existing data regarding the association between MAU and stroke type are controversial. For example, Beamer et al. found MAU differed among major ischemic stroke subtypes [9]. In contrast, it has been shown that MAU is related to carotid atherosclerosis [23,24] and these findings corroborate our results of MAU being associated with large-artery stroke.

### MAU as an indicator of atherosclerosis

There is a significant association between microalbuminuria and carotid artery intima-media thickness, which suggests that MAU may be a marker for early development of carotid artery atherosclerosis and points to a possible linkage between microalbuminuria and an atherothrombotic stroke mechanism [2,24-26]. In a study by Ravera et al. MAU was associated with a higher carotid IMT (0.94 ± 0.05 vs. 0.75 ± 0.06 mm; p = 0.03) compared with normoalbuminuric patients with untreated essential hypertension [2]. Furtner et al. previously reported a significant correlation of carotid artery intima-media thickness with MAU [25]. Recent research likewise suggested that albuminuria is associated with increased carotid IMT in Chinese type-2 diabetic patients even below the traditional cut-off values for microalbuminuria (low-grade albuminuria) [27]. The results of our analyses confirm these findings in patients after recent stroke. These associations persisted even after adjusting for differences in baseline characteristics.

MAU has previously been shown to be associated with PAD, more so in non-diabetic than in diabetic patients [28]. Escobedo et al. recently reported that MAU is an important predictor of peripheral artery disease (defined by an ABI ≤ 0.9) in CAD patients from the BARI 2D study [29]. Our data demonstrate that the detection of MAU is independently associated with an ABI < 0.9 even in patients with stroke.

To the best of our knowledge the present study analysed the association between polyvascular disease (defined as increased IMT and ABI < 0.9) and MAU for the first time in stroke patients. We observed a significantly increased prevalence of polyvascular disease with an RR of 1.56 in patients with MAU. It is well known (e.g. from the REACH registry data) that polyvascular disease is related to an increased risk of future vascular events, hospitalisation and mortality [30-32]. Thus, our data implicate, that the detection of MAU in stroke patients is an indicator for more advanced atherosclerosis in different vascular beds and an increased vascular risk.

### MAU as a predictor of vascular events within 1 year after stroke

The ability of MAU to identify patients at high risk for myocardial infarction, stroke and all cause mortality has been described in a number of cross-sectional and prospective studies, which also usually documented a positive correlation between the degree of albuminuria and the severity of the event [3,5,6,33]. MAU is a frequent finding in several acute cardiovascular events including stroke and is an independent predictor of 1-year mortality following acute ischemic stroke [9-11]. In the Tromso Study [6] a high urinary albumin-creatinine ratio (ACR) was associated with an increased risk of myocardial infarction, stroke, and all-cause mortality after a mean follow-up about 10 years. Multivariable adjustment revealed, that this was not related to the presence of the metabolic syndrome.

In the present study, patients with sub-acute stroke and MAU had a more than doubled risk of cardiovascular events including non-fatal stroke or MI and fatal events (HR 2.2; 95%CI 1.3-3.7 for total mortality) even after adjusting for several baseline parameters, risk factors and previous vascular disease. While specificity of MAU testing was high, sensitivity was low. This may hint at the necessity of repeated testing as suggested in a number of guidelines [34].

### Limitations

Despite the large number of patients included in our case series from 15 neurologic rehabilitation centres in Germany, the present analysis is limited by the fact that 1) We included stroke patients from neurologic rehabilitation. This may have led to a selection bias towards more severe stroke patients due to the German specialty of sending patients with more severe acute stroke deficits for to in-hospital rehabilitation. 2) Follow-up information was obtained from only 72.5% of patients. On the other hand, the baseline data between the patients without follow-up are very similar to the patients with follow-up with no significant differences except for age. Therefore a relevant bias seems unlikely. 3) Follow-up was only 13 months. 4) MAU determination was conducted using the Microalbustix® that measures albuminuria and creatinine only semi quantitatively. While the bias introduced is negligible in many patients it cannot be ruled out that some where misclassified. It was recently shown however, that dipstick screening of microalbuminuria could be used in a population at risk of chronic kidney disease [35]. 5) We obtained no information on blood glucose or HbA1c. 6) Follow-up information including primary and secondary endpoints has been collected only by telephone and in case of death or inability of the patient to communicate via family members or other third persons. No on-site visit was performed to check validity of obtained outcome data.

## Conclusions

INSIGHT demonstrated a significant association between MAU and polyvascular disease and further supports previous findings that MAU predicts cardio-/cerebrovascular events in patients recovering from ischemic stroke. This biomarker may also be used in patients during neurologic in-patient rehabilitation, opening a window of opportunity for early intervention in this patient group at increased risk for recurrent events.

## Competing interests

DS, CW, and PB declare to have received research support/lecture honoraria from Sanofi Aventis. LR is an employee of Sanofi Aventis. All other authors report no conflict.

## Authors' contributions

PB, LR and DS designed the study; MS, CW, TB, DS acquired the data; PB and DS analysed and interpreted the data. PB and DS drafted the article and all authors revised the manuscript for important intellectual content. All authors approved the final manuscript.

## Pre-publication history

The pre-publication history for this paper can be accessed here:

http://www.biomedcentral.com/1471-2377/12/102/prepub
